# Orally Administered, Biodegradable and Biocompatible Hydroxypropyl–β–Cyclodextrin Grafted Poly(methacrylic acid) Hydrogel for pH Sensitive Sustained Anticancer Drug Delivery

**DOI:** 10.3390/gels8030190

**Published:** 2022-03-19

**Authors:** Nighat Batool, Rai Muhammad Sarfraz, Asif Mahmood, Muhammad Zaman, Nadiah Zafar, Ahmad Salawi, Yosif Almoshari, Meshal Alshamrani

**Affiliations:** 1 Faculty of Pharmacy, College of Pharmacy, University of Sargodha, Sargodha 40100, Pakistan; nighatbatool2352@gmail.com; 2 Department of Pharmaceutics, Faculty of Pharmacy, The University of Lahore, Lahore 54000, Pakistan; nadiah.zafar@pharm.uol.edu.pk; 3 Faculty of Pharmacy, University of Central Punjab, Lahore 54000, Pakistan; dr.mzaman@ucp.edu.pk; 4 Department of Pharmaceutics, College of Pharmacy, Jazan University, Jazan 45142, Saudi Arabia; asalawi@jazanu.edu.sa (A.S.); yalmoshari@jazanu.edu.sa (Y.A.); malshamrani@jazanu.edu.sa (M.A.)

**Keywords:** cytarabine, hydroxypropyl–β–cyclodextrin, pharmacokinetics, half-life, colorectal cancer

## Abstract

In the current study, a pH sensitive intelligent hydroxypropyl–β–cyclodextrin-based polymeric network (HP-β-CD-g-MAA) was developed through a solution polymerization technique for site specific delivery of cytarabine in the colonic region. Prepared hydrogel formulations were characterized through cytarabine loading (%), ingredient’s compatibility, structural evaluation, thermal integrity, swelling pattern, release behavior and toxicological profiling in rabbits. Moreover, the pharmacokinetic profile of cytarabine was also determined in rabbits. New polymer formation was evident from FTIR findings. The percentage loaded into the hydrogels was in the range of 37.17–79.3%. Optimum swelling ratio of 44.56 was obtained at pH 7.4. Cytarabine release was persistent and in a controlled manner up to 24 h. In vitro degradation of hydrogels was more pronounced at intestinal pH as compared to acidic pH. Toxicity studies proved absence of any ocular, skin and oral toxicity, thus proving biocompatibility of the fabricated network. Hydrogels exhibited longer plasma half-life (8.75 h) and AUC (45.35 μg.h/mL) with respect to oral cytarabine solution. Thus, the developed hydrogel networks proved to be excellent and biocompatible cargo for prolonged and site-specific delivery of cytarabine in the management of colon cancer.

## 1. Introduction 

For years researchers have been trying to develop target-based drug delivery systems (DDS) owing to their propensity to alleviate adverse effects and optimize therapeutic efficacy. Novel sustained release drug delivery systems are designed to control the drug concentration at the target site, reduce toxicity, optimize efficacy and provide higher patient compliance. A number of dosage forms have been developed for controlled drug delivery, such as liposomes, nano-technology, polymeric micelles, polymer-drug conjugates, dendrimers, films and hydrogels [1,2].

Hydrogels are considered as swellable systems which are most appropriate for site-specific sustained delivery of several drugs. They are versatile, three dimensional, crosslinked polymeric networks fabricated through solution polymerization/cross linking, bulk polymerization, free-radical polymerization, suspension or inverse-suspension polymerization and polymerization by irradiation techniques [3,4]. Chemically crosslinked hydrogels exhibit pronounced swelling when compared to radiation induced crosslinked hydrogels. Their potential to respond to environmental stimuli, such as pH, temperature, electric field and ionic species, has grabbed the attention of researchers since the last decade. In pH sensitive hydrogels, presence of hydrophilic groups, such as –OH, –COOH, –SO_3_H, –CONH_2_ and –CONH, impart swelling and deswelling character in response to pH stimulus [5]. Exposure to media of specific pH induces generation of ionized groups which promotes volume transitions in terms of swelling due to repulsion between ionized groups [6,7]. 

Cytarabine (cytosine arabinoside or 1-β, D-arabino-furanosyl cytosine) was chosen as a typical anticancer drug. It is employed in treatment of different cancers, such as myeloid leukemia, colorectal cancer and non-Hodgkin’s lymphoma. It belongs to BCS class III. It has a short plasma half-life of 10–20 min [8], and low oral bioavailability of about 20% [9]. The standard dose of cytarabine used to treat acute leukemia is 3 g/m^2^ through I/V route every three hours. Being water soluble, cytarabine undergoes a rapid metabolism by cytidine de-aminase to an inactive metabolite, 1-β, D-arabino-furanosyl uracil. This oxidative reaction reduces the activity of cytarabine and the resultant metabolite is eliminated rapidly through urine as uracil arabinoside [10].

HPβCD is a hydrophilic and safe polymer with low renal toxicity. It improves functionality and solubility of drugs having low permeability (e.g., cytarabine) owing to its lipophilic inner core. It acts as a penetration enhancer and solubility modifier for lipophilic drugs [11,12]. In literature, it is evident that introduction of HPβCD to numerous drug delivery systems, such as buccal, transdermal and ocular, promotes solubility and bioavailability of incorporated less polar dugs [13,14]. HPβCD is a derivative of β-cyclodextrin (βCD) obtained by structural modifications of βCD by substituting hydroxyl (-OH) functional groups at positions C_6_, C_2_ or C_3_. HPβCD has also been used for oral controlled delivery of different therapeutic agents. Methacrylic acid (MAA) being a monomer is pH sensitive and exhibits extraordinary swelling properties in basic environments. Methylene bis-acrylamide (MBA) is a cross linker and it possesses excellent gelation properties through physical interactions [15,16,17]. Ammonium per sulphate (APS) is an initiator that is used to generate active sites at the polymer backbone and hence initiates polymerization reaction. 

The aim of this study was to develop hydroxy propyl β-cyclodextrin based polymeric hydrogels with a focus on improving bioavailability of cytarabine through the oral route, thereby prolonging its half-life, reducing its dosing frequency and hence improving patients’ compliance. Moreover, the study also aimed to evaluate the effect of formulation contents on different parameters of developed hydrogels, such as swelling studies, gel fraction, release and pharmacokinetic profile of cytarabine. 

## 2. Results and Discussion

### 2.1. FTIR Analysis

**Figure 1:**
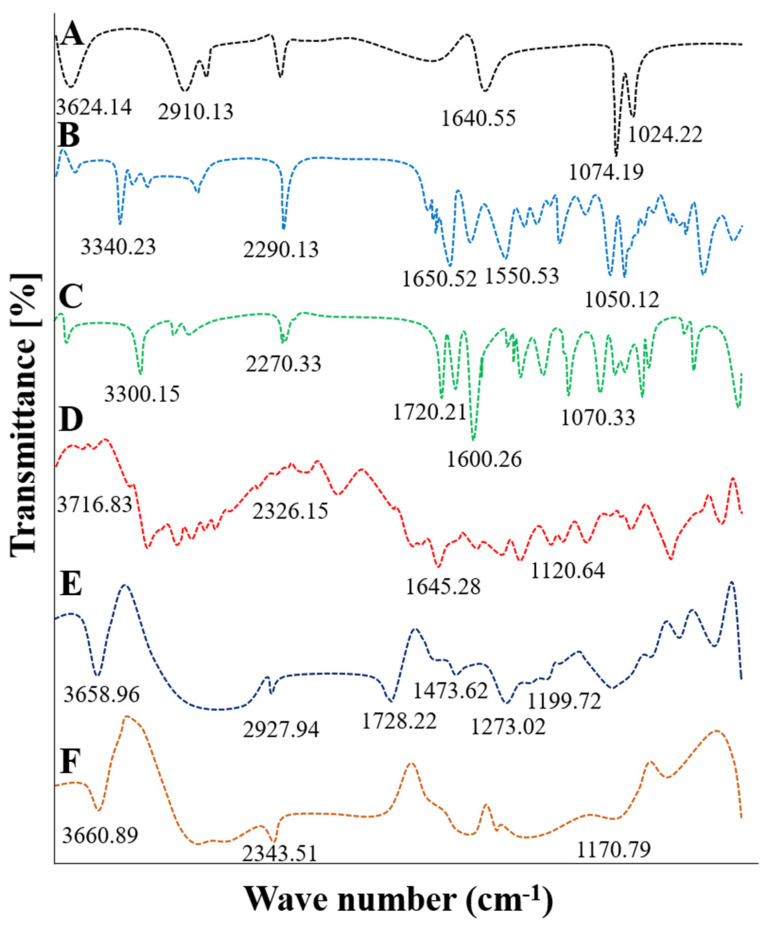
FTIR spectra of HPβCD (**A**), cytarabine (**B**), MBA (**C**), physical mixture (**D**), unloaded hydrogels (**E**) and cytarabine-loaded hydrogel network (**F**).

FTIR spectra of pure cytarabine, HPβCD, blank and cytarabine-loaded HPβCD hydrogel along with their physical mixture were obtained and compared (Figure 1).

FTIR spectrum of HPβCD showed prominent peaks at 3624.14 cm^−^^1^ and 2910.13 cm^−1^ due to free –OH and –CH_3_ stretching vibrations (Figure 1A). FTIR spectrum of pure cytarabine powder demonstrated absorption peaks at 1050.12 cm^−^^1^, 3340.23 cm^−^^1^, 1650.52 cm^−^^1^, 1550.53 cm^−^^1^ and 2290.13 cm^−^^1^ corresponding to stretching vibrations of –CO, –NH, C=O, –CN and –CH groups, respectively (Figure 1B). MBA showed prominent peaks at 3300.15 cm^−1^, 2270.33 cm^−1^, 1720.21 cm^−1^, 1600.26 cm^−1^ and 1077.13 cm^−1^ due to C=O and N-H groups (Figure 1C). Absence of occurrence of any new peak in IR spectrum of physical mixture confirmed (Figure 1D) the compatibility of used ingredients. 

FTIR spectrum of developed blank hydrogel (Figure 1E) presented typical peaks at 3658.96 cm^−^^1^ and 2927.94 cm^−^^1^ due to stretching movements of the –OH and –CH_2_ functional groups of the HPβCD. Peaks observed at 1728.22 cm^−^^1^ due to H–O–H bending vibrations and at 1473.62 cm^−^^1^ and 1273.02 cm^−^^1^ due to –COO symmetrical stretching movements of –COO and –CO functional groups verified MAA crosslinking with polymeric backbone. While a peak at 1199.72 cm^−1^ revealed ester sulphate stretching of ammonium per sulphate, thus confirming new polymeric hydrogel formation comprised of HPβCD as polymer backbone. Our results can be compared to previous literature where peaks disappeared or shifted in HPβCD based nano gels in comparison to pure ingredients [17]. In FTIR spectrum of cytarabine-loaded hydrogel, the absorption band of pure cytarabine at 1170.79 cm^−^^1^ shifted to 1050.12 cm^−^^1^ due to the existence of a carbonyl group. The peak due to amino group stretching at 3340.23 cm^−^^1^ was shifted to 3660.89 cm^−^^1^ confirming the presence of –NH species. The C=O stretching was observed at 2343.51 cm^−^^1^ instead of 1650.52 cm^−^^1^ (Figure 1F) [18].

### 2.2. Energy Dispersive X-ray Analysis (EDX)

**Figure 2:**
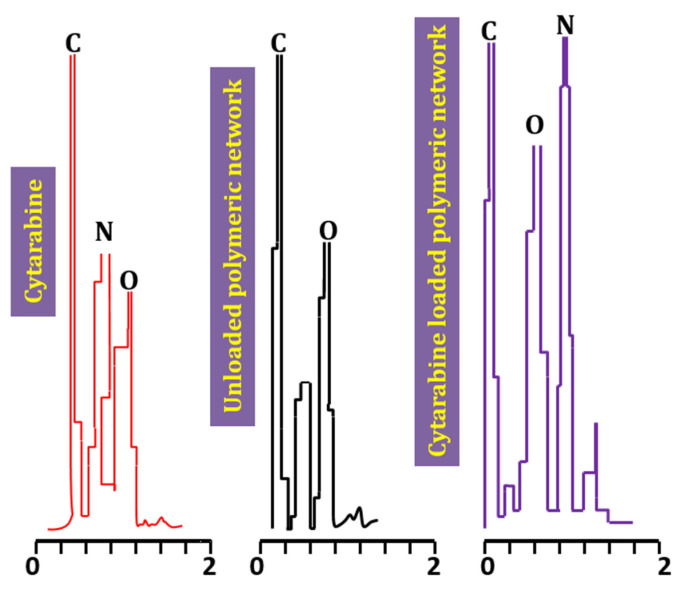
EDX spectra of pure drug, Unloaded hydrogels and loaded hydrogels.

Percent weights of carbon, oxygen and nitrogen identified in pure cytarabine powder were 60.18%, 27.93% and 11.9%. The blank hydrogel contained carbon (50.33%), oxygen (25.14%) and nitrogen (1.2%) while in the cytarabine-loaded hydrogel these contents were 69.96%, 48.47% and 4.9% for carbon, oxygen and nitrogen, respectively. As nitrogen is an essential element of cytarabine, its peak was negligible or absent in the blank hydrogel. In the cytarabine-loaded hydrogel, the percent weight of C, O and N was increased compared to the blank hydrogel (Figure 2). Similar findings were observed in a study conducted by Ibrahim et al. (2019) highlighting a rise in percent weight of elements in the drug-loaded hydrogel during synthesis of polyacrylamide-grafted chitosan hydrogels [18].

### 2.3. Swelling Studies

**Figure 3:**
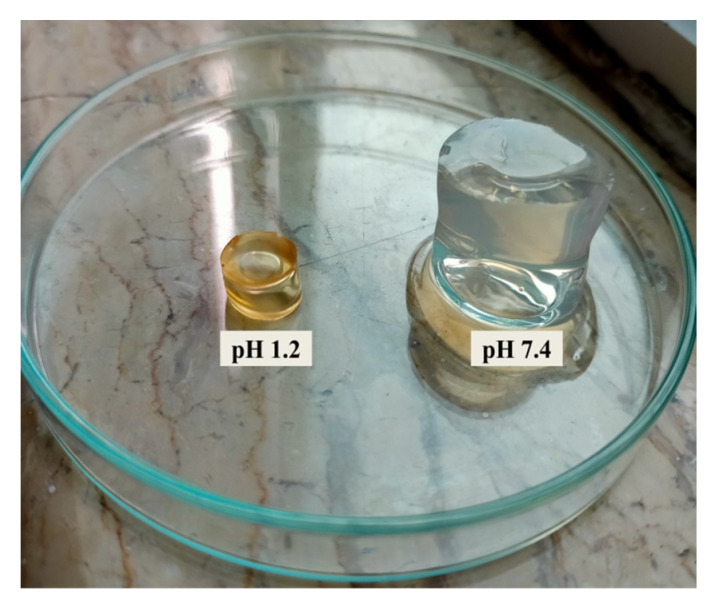
pH dependent swelling of HPβCD-grafted poly(MAA) hydrogels.

Developed hydrogels were pH responsive exhibiting pronounced swelling at higher pH value (7.4) compared to low pH value (1.2) as shown in Figure 3.

**Figure 4:**
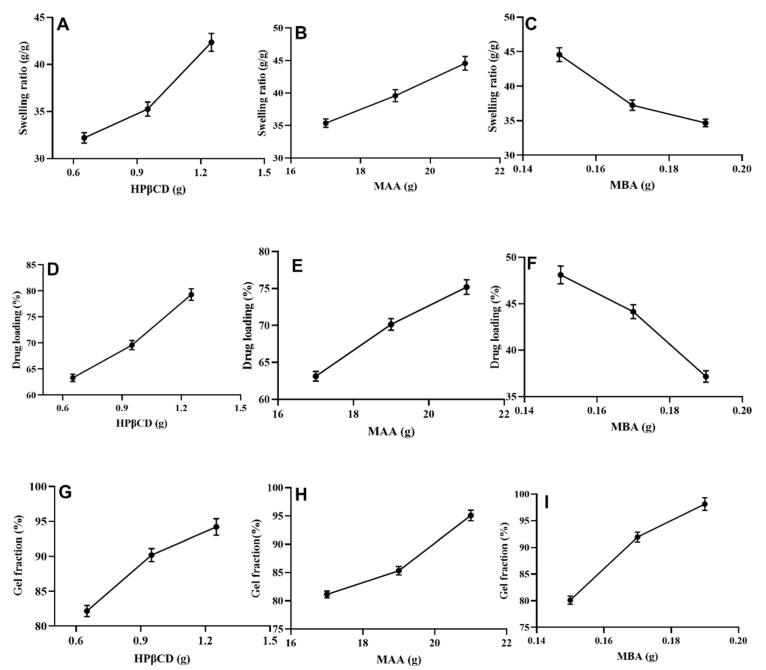
Effect of HPβCD on swelling ratio (**A**), % drug loading (**D**), and % gel fraction (**G**), effect of MAA on swelling ratio (**B**), % drug loading (**E**), % gel fraction (**H**), effect of MBA on swelling ratio (**C**), % drug loading (**F**) and % gel fraction (**I**).

An increase in swelling ratio of the developed hydrogel was observed with increasing contents of HPβCD (32.19–42.34 for HPM-1–HPM-3 and MAA (35.36–44.56) for HPM-7–HPM-9 as shown in Figure 4A, Figure 4B, respectively. Similar findings were obtained by Diez et al. (2002) where the formed hydrogel demonstrated more swelling with increasing contents of MAA and hydroxy propyl β-cyclodextrin [19]. Hydrophilic property and porous morphology of HPβCD was considered to be responsible for this increased swelling. Furthermore, ionization of hydroxyl groups of HPβCD and –COOH group of MAA at alkaline pH caused more water uptake leading to increased swelling [20]. 

A decrease in swelling ratio was observed with increasing amount of methylene bisacrylamide for formulations HPM-4–HPM-6 (Figure 4C). This decrease in swelling ratio was attributed to higher crosslinking density. This finding is in agreement with the results of Kabiri et al. (2003) and Anirudhan et al. (2018) where a decrease in swelling capacity of prepared hydrogel was noted with an increase in MBA contents [21,22].

### 2.4. Loading of Cytarabine (%)

Highest drug loading (79.3%) was noticed for formulation HPM-3 (Figure 4D) with maximum concentration (1.25 g) of HPβCD. A high drug loading was also observed for formulation HPM-9 (75.19%) with highest amount (21 g) of methacrylic acid (Figure 4E). HPβCD and MAA get ionized at pH of the drug solution. These ionized groups display electrostatic repulsions, swelling and hence lead to higher drug loading. Whereas, minimum drug loading (39.17%) was observed in formulation HPM-6 which contained 0.19 g of MBA (Figure 4F). This low loading efficiency was observed because of the dense polymeric network which hindered greater fluid penetration.

Our results are in agreement with previous studies where drug loading increased with increasing quantities of polymer and monomer but a rapid decline in drug loading was observed by increasing concentration of cross linker [23].

### 2.5. Sol-Gel Fraction 

Overall gel content was increased in all formulations (HPM-1–HPM-9) with increasing concentration of all used ingredients. Formulations (HPM1–HPM3) with increasing contents of HPβCD (0.65–1.25 g) exhibited gel fractions ranging from 82.17 to 94.21% (Figure 4G). Formulations (HPM4–HPM6) with increasing MAA contents (17–21 g) also showed significant gradual rise in gel fraction, i.e., from 81.14 to 95.10% (Figure 4H) but with an increase of MBA contents (0.15–0.19 g) optimum rise in gel fraction, i.e., 80.12–98.13% was observed (Figure 4I). These findings are supported by another study conducted by Minhas et al. (2016), i.e., a rise in gel content with the incremental increase of polymer, monomer and cross-linker contents [24]. 

### 2.6. DSC Studies

**Figure 5:**
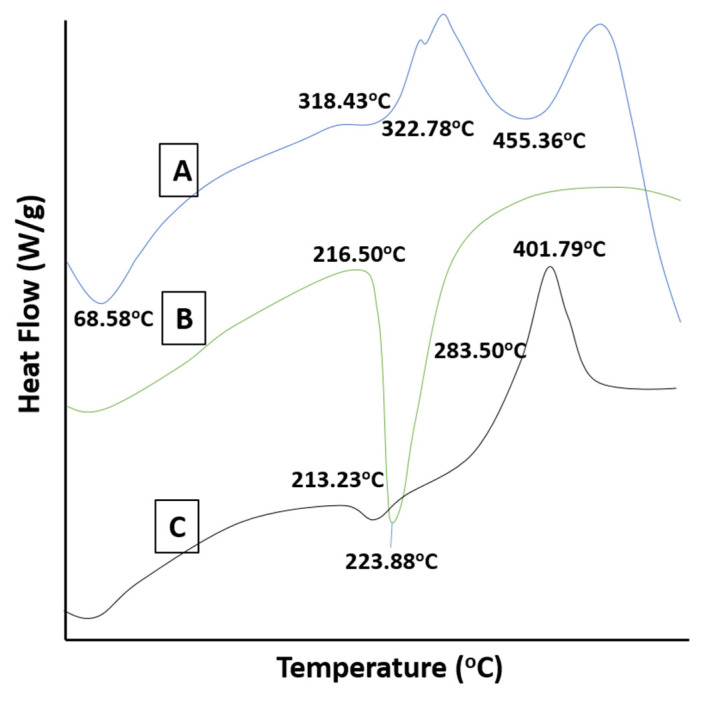
DSC thermograms of HPβCD (A), cytarabine (B) and cytarabine-loaded hydrogels (C).

DSC thermogram of HPβCD exhibited an endothermic peak at 68.58 °C, most probably due to moisture loss from the polymer. Another broad endothermic peak appeared at 455.36 °C indicating complete combustion of HPβCD (Figure 5A). For cytarabine, the diffused peak at 216.50 °C was attributed to evaporation of moisture and an endothermic peak appeared at 223.88 °C representing the melting of the drug (Figure 5B). The DSC thermogram of the cytarabine-loaded hydrogel displayed a sharp exothermic peak at 401.79 °C confirming the presence of cytarabine in the developed hydrogel system (Figure 5C). Our results are in agreement with Deepa et al. (2018) where loss of the typical peak of the active drug and appearance of new peak patterns within range of 250–260 °C confirmed uniform distribution of cytarabine (amorphous state) in the chitosan-based hydrogel [25]. 

### 2.7. TGA Studies

**Figure 6:**
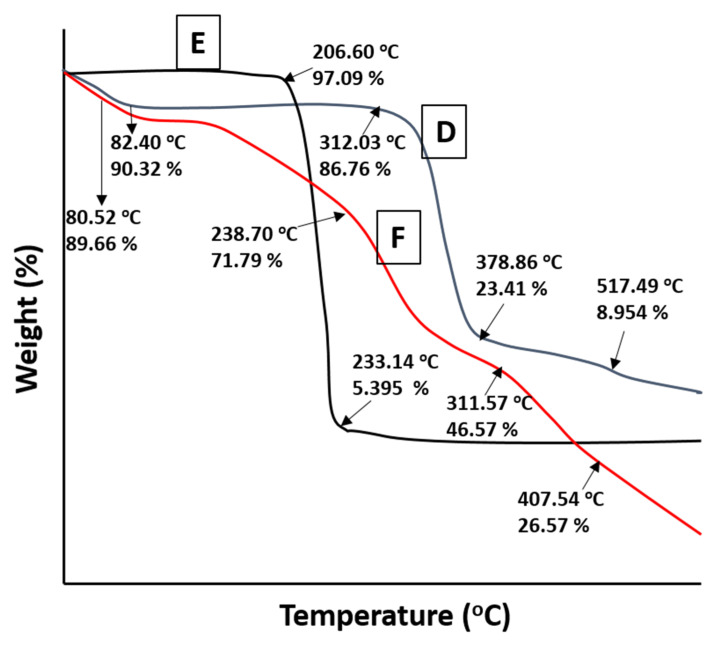
TGA thermograms of HPβCD (D), Cytarabine (E) and Cytarabine-loaded hydrogels (F).

Thermogravimetric analysis (Figure 6D) showed loss of weight of HPβCD, cytarabine and the newly developed hydrogel as the temperature was increased. The thermogram of pure HPβCD showed 9.68%, 77.59% and 90% weight loss at 82.40 °C, 378.86 °C and 517.49 °C, respectively, due to moisture escape and polymer degradation. The TGA graph of the drug showed 2.87% and 94.60% mass loss at 206.60 °C and 233.14 °C, respectively, because of moisture loss and cytarabine decomposition (Figure 6E). In the TGA thermogram of the cytarabine-loaded hydrogel network, weight loss of 11.33%, 29.21%, 54.43% and 74.44% occurred at 80.52 °C, 238.70 °C, 311.57 °C and 407.54 °C, respectively, thereby confirming stability of cytarabine within the developed network (Figure 6F). Previously, similar findings were reported by Khatun et al. (2020) where shifting of peaks to higher decomposition temperature confirmed higher thermal stability of curcumin-grafted-HPβCD polymerized hydrogels [20].

### 2.8. Scanning Electron Microscopy

**Figure 7:**
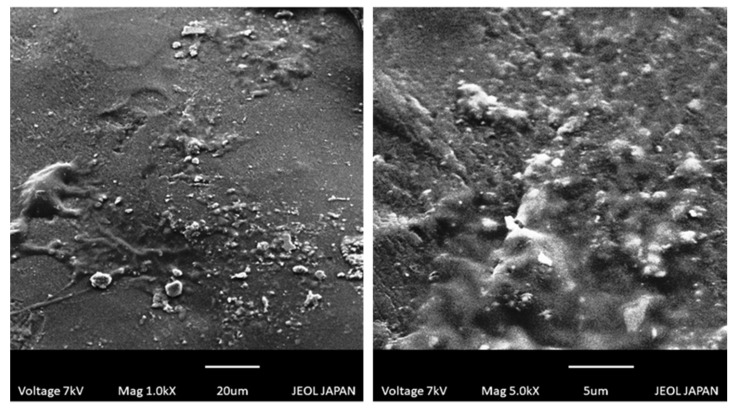
SEM photomicrographs of developed hydrogels.

SEM studies revealed that developed hydrogels had rough surfaces with smooth texture (Figure 7). All the developed hydrogel formulations had glossy, rubbery and elastic appearance. Raza et al. (2017) also reported rough and flakey surfaces of HPβCD-based hydrogel after drug incorporation, which are similar to our observations [26]. 

### 2.9. Powdered X-ray Diffraction Studies 

**Figure 8:**
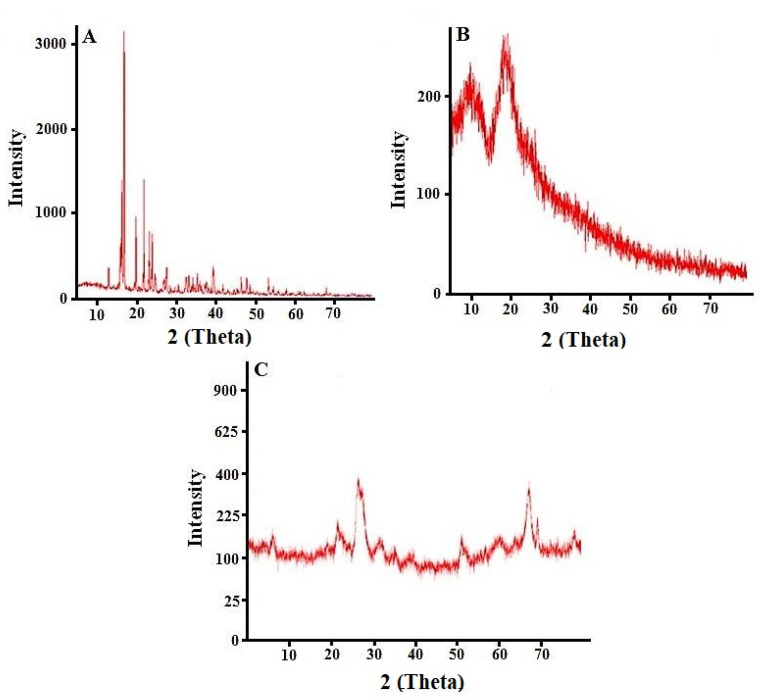
PXRD diffractogram of cytarabine (**A**), HPβCD (**B**) and cytarabine-loaded hydrogel network (**C**).

The cytarabine scan presented a specific pattern in the XRD findings with peaks at different angles, i.e., 17.5°, 19.5°, 22.4° and 24.3° indicating its crystalline nature (Figure 8A) whereas the XRD diffractogram of pure HPβCD confirmed its amorphous nature (Figure 8B). XRD diffractogram of cytarabine encapsulating hydrogel (Figure 8C), showed a lack of major peaks of cytarabine indicating loss of crystallinity of drug as well as successful incorporation of cytarabine into developed HPβCD-based hydrogels.

Similar observations were made by Eleamen et al. (2017) who noticed a change of crystalline behavior of the drug to an amorphous form, during improvement of solubility and antifungal activity of aminothiophene derivative with 2-hydroxypropyl-β-cyclodextrin [27]. 

### 2.10. Tensile Analysis

**Figure 9:**
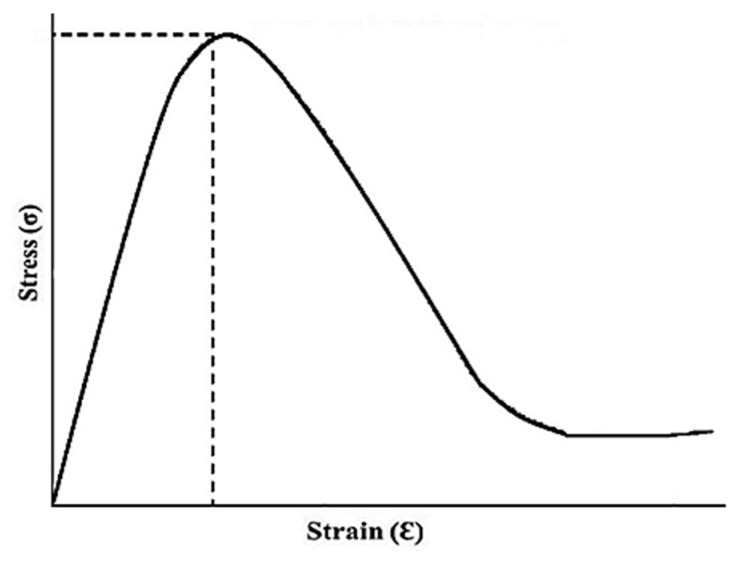
Tensile stress–strain curve.

The fabricated hydrogel network showed better mechanical power with tensile strength of 41.514 N/mm^2^, Young’s modulus of 356.24 N/mm^2^ and percent elongation of 36.09%. The load at time of break was observed to be 5.610 N. Results are shown in Figure 9. The surface of fabricated hydrogel remained uniform in all formulations throughout the process. This improvement of mechanical properties was due to greater concentration of HPβCD and MBA contents [28].

### 2.11. Drug Release and Kinetic Modelling

**Figure 10:**
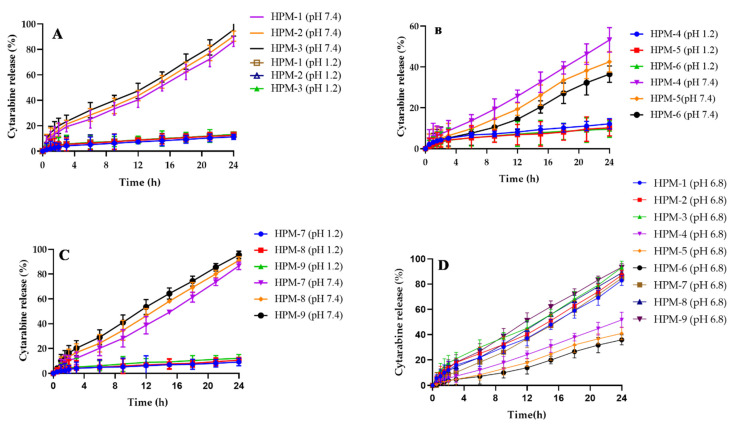
Cytarabine release studies at pH 1.2 and 7.4. Effect of HPβCD on cytarabine release (**A**), effect of MBA on cytarabine release (**B**), effect of MAA on cytarabine release (**C**) and cytarabine release at intestinal pH (**D**).

Drug release studies (Figure 10) showed different release patterns for cytarabine depending on different components of the formulated hydrogels. Cytarabine release of 86.17–95.47% was measured with increasing content of HPβCD in the developed hydrogel formulations (HPM-1–HPM-3) (Figure 10A). Increase in drug release of 86.67–95.57% (HPM-7–HPM-9) (Figure 10C) was also noticed with an increase of MAA contents when other constituents’ concentrations remained constant. Improvement in drug release with increased concentration of HPβCD and methacrylic acid during preparation of the pH-sensitive hydrogel has already been reported in literature [29,30].

**Figure 11:**
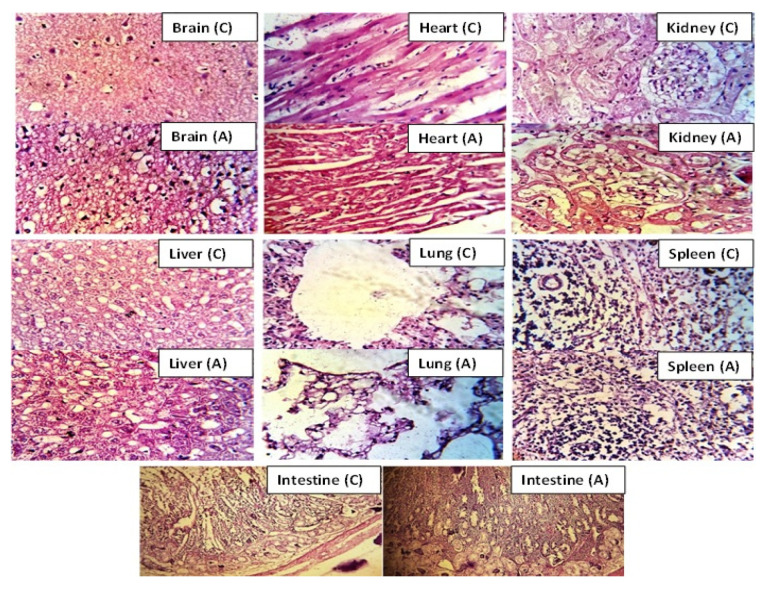
Histopathological images of vital organs of rabbit after administration of hydrogel network (2 g/kg) in control group (C) and treated group (A).

A decline in release of cytarabine from 53.21 to 36.49% for formulations (HPM-4–HPM-6) was observed when concentration of MBA was increased (Figure 10B). Rehman et al. (2021) reported a decrease in drug release with increasing contents of MBA [31]. Cytarabine release patterns were also observed at pH 6.8. A similar trend as observed at pH 7.4 but with slight decline in cytarabine release, i.e., rise in cytarabine release (82.97–93.22%) and (84.67–93.42%) with respect to HPβCD (0.60–1.25 g) and MAA (15–19 g) contents, respectively, was noted while a decrease in cytarabine release (51.71–35.99%) was noted in a parallel manner by increasing MBA (0.15–0.19 g) contents. Results are shown in Figure 11D.

All the developed formulations followed the zero-order release model with R^2^ values within 0.9759–0.9991 (Table 1)**.** The release mechanism for cytarabine was found to be Super Case II transport based on value of “*n*”, i.e., more than 0.89. 

### 2.12. Acute Oral Toxicity Evaluation 

All rabbits were carefully examined, to evaluate oral toxic effects of newly prepared cytarabine-loaded hydrogel, regarding diet and water intake, body weight, diarrhea, irritation, any illness, ocular and dermal toxicity throughout the period of toxicity studies. All investigational animals were properly weighed before and after treatment. 

Physical parameters were intact with no signs of visual or skin toxicity. Moreover, no death of the animal was reported throughout investigation. Results are computed in Table 2, Table 3 and Table 4. 

#### Histology Studies

After 14 days of treatment, vital organs from rabbits of control and treated groups were removed and weighed after being sacrificed. No difference was seen in weights of vital organs of both control and treated groups. No mark of disease, soreness or abnormality was observed during tissue examination of key organs under light microscope (40×). Tissues were suitably organized with no sign of injury. Death rate was zero throughout the toxicity study. 

Myocytes cells of heart tissue were accurately arranged with original cellular geometries without any sort of destruction or inflammation. Liver tissues of both control and administered animals were normal with properly arranged hepatic cord and lobules. Lymphocytes, macrophages, monocytes and neutrophils were also organized normally with no inflammation. Lungs and their bronchioles were also normal. Microscopic evaluation of rabbit’s renal tissue presented cells with nuclei. No sign of cellular destruction, presence of blood or blood clot was seen in the brain and intestinal tissues. Similarly, cells of the spleen remained unaffected in terms of their shape in all groups treated with HPβCD-grafted hydrogel network (Figure 11). Tan et al. (2013) used all parameters as in our studies and performed oral toxicity based on caprolactone and itaconic acid pH sensitive hydrogel using mice as animal model [32]. Results were similar to our observations. 

### 2.13. In Vivo Studies

**Figure 12:**
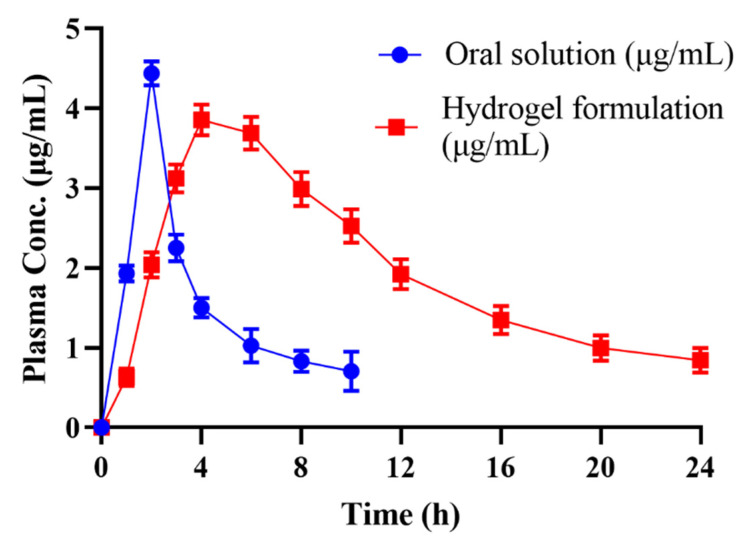
Combined plasma concentration graph of pure cytarabine powder solution and cytarabine-loaded hydrogel formulation after oral administration.

The amount of cytarabine within plasma with the passage of time was scrutinized using a validated HPLC method. Plasma versus time results after oral administration of the pure drug powder and equivalent drug-loaded networks are presented in Figure 12. 

The pure drug achieved optimum plasma level (C_max_ = 4.43 μg/mL) within 2 h followed by its decline to zero within 10 h. C_max_ in the case of the drug-loaded carrier system (C_max_ = 3.85 μg/mL) was attained in 4 h and was reflected in plasma for 24 h. 

Results of pharmacokinetics are mentioned in Table 5. Half-life of cytarabine within polymeric network was higher (t_1/2_ = 8.75 h) than the value observed for cytarabine powder solution (t_1/2_ = 4.13 h). MRT in the case of cytarabine was significantly increased (MRT = 14.93 h) when the drug was administered within the developed carrier rather than cytarabine powder solution (MRT = 6.43 h). These findings clearly proved that the developed polymeric device possessed excellent slow releasing potentials. 

Zhang et al. (2016) performed pharmacokinetic evaluation of cytarabine and its colic acid conjugates in rats. Administered dose of cytarabine and its cholic acid conjugates was 30 mg/kg orally [33]. Observed pharmacokinetic parameters AUC _(0-inf)_ (16,603.7–34,857.0 ng·h/mL), C_max_ (2103.4–1706.1 ng/mL), t_max_ (2.5–3.6 h) and elimination half-life was in range of 4–15.62 h for cytarabine and cytarabine colic acid conjugates which can be correlated to our observed values. 

### 2.14. In Vitro Degradation Studies 

**Figure 13:**
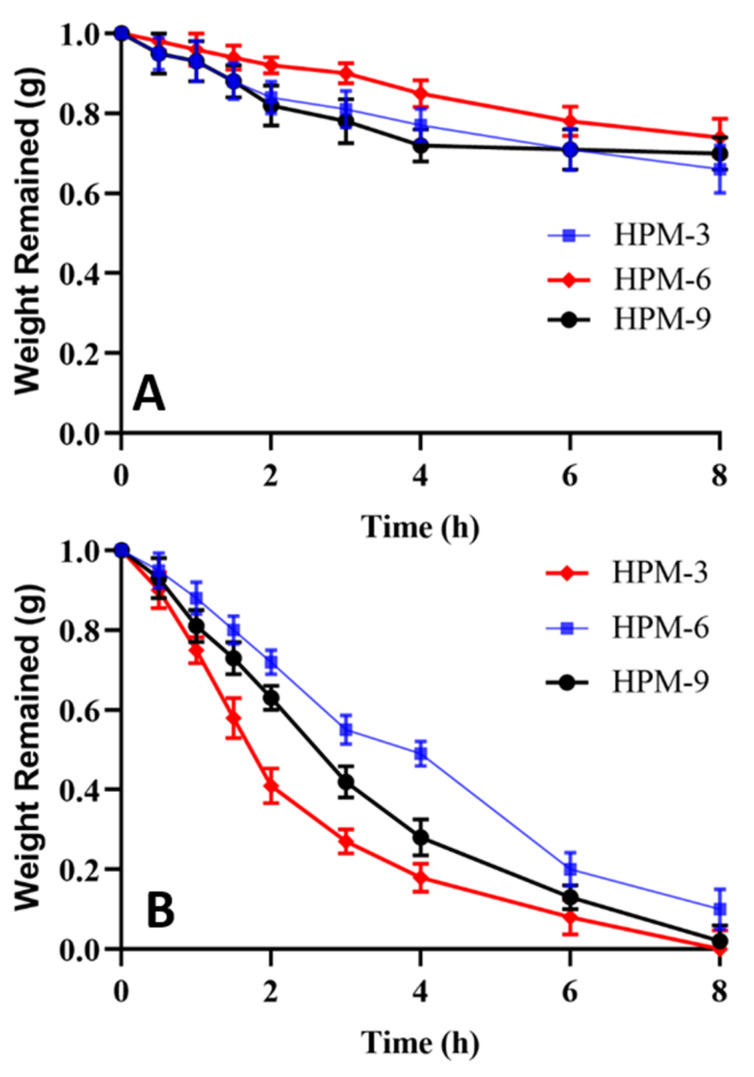
In vitro degradation studies at (**A**) AGF (pH 1.2) and (**B**) AIF (pH 6.8).

Developed hydrogels exhibited variable degradation behavior in AGF (pH 1.2) and AIF (pH 6.8). At pH 1.2, hydrogels presented hydrophobic character due to lack of ionization of carboxylic groups of MAA and hence minimal degradation occurred. Formulation having optimum MBA content (HPM-6) showed only 0.26 g weight loss in 8 h studies at AGF. On the other hand, slightly higher weight loss was recorded in the case of the formulation (HPM-9) having optimum content of MAA. While in the case of HPβCD (HPM-3) weight loss was only 0.34 g. Results are shown in Figure 13A. These results suggested that as there was negligible swelling at acidic pH, thus the least weight was wasted and maximum weight remained intact. 

At intestinal pH (6.8), carboxylic groups get ionized into carboxylate ions that result in imbibition of media and hence swelling of the network. In AIF, degradation was rapid. Results are shown in Figure 13B. All test formulations, HPM-3, HPM-6 and HPM-9, exhibited degradation but the rates of degradation were in the order of HPM-3 > HPM-9 > HPM-6. Rapid degradation is associated with weak interactive forced between polymeric chains. 

## 3. Conclusions

HPβCD and methacrylic acid were successfully crosslinked to develop a pH sensitive hydrogel system. The prepared hydrogel network showed ingredients’ compatibility as confirmed by FTIR and thermal integrity proven through DSC and TGA studies. SEM findings confirmed the smooth surface of fabricated hydrogel with presence of holes and cracks. EDX showed elemental composition of hydrogel. The highest drug loading (79.3%) was observed for formulation HPM-3 with the highest concentration of HPβCD and it also showed maximum swelling ratio and drug dissolution at pH 7.4. Developed formulations demonstrated controlled drug release pattern in the range of 36.49–95.97%. An optimized formulation, HP-7, was chosen for toxicity and in vivo studies of fabricated hydrogel in rabbits. Histopathological observations did not show any symptoms of abnormality or pathological distortion. In vivo pharmacokinetic studies demonstrated prolonged t_1/2_, increased MRT and AUC of cytarabine following oral delivery of the prepared hydrogel. Thus, the developed HPβCD-based hydrogel system is safe for oral administration of anti-cancer drugs.

## 4. Material and Methods

### 4.1. Materials

Hydroxy propyl β-cyclodextrin (99.9%), methacrylic acid (99.88%), ammonium per sulfate (99.97%), N, N-methylene bisacrylamide (MBA) (99.8%), n-hexane (99.81%) and ethanol (99.84%) were purchased from Sigma-Aldrich Co., St Louis, MO, USA. Buffering agents were obtained from Merck Germany. All the chemicals consumed in the current study were of analytical grade. 

### 4.2. Development of HPβCD-Grafted-Poly (MAA) Polymeric Networks

**Figure 14:**
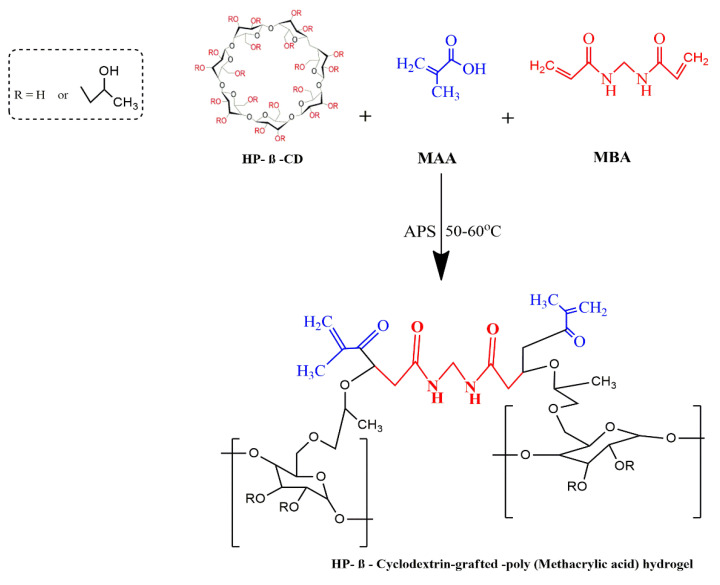
Schematic presentation of developed HPβCD-grafted-poly (MAA) hydrogels.

Aqueous polymerization involving free radical exchange was used to develop various hydrogel preparations. A weighed amount of HPβCD (Table 6) was dissolved in 25 mL freshly prepared distilled water with constant stirring at room temperature on a hot plate. A pre-weighed quantity of ammonium per-sulfate (APS) was dissolved in a specific quantity of water and poured dropwise into the HPβCD solution while on the hot plate stirrer. An amount (Table 1) of methacrylic acid (MAA) was added drop wise to the above mixture. Finally, an aqueous solution of MBA was transferred slowly to the polymer-monomer mixture. The resultant solution was sonicated for at least 3–5 min to remove entrapped air. The clear solution was transferred to test tubes which were closed with aluminum paper. Test tubes were vertically racked on a steel test tube stand and the entire setup was translocated into a water bath. Initially the temperature was kept at 45 °C for 2 h followed by incremental rises in temperature, i.e., 50 °C for 2 h and finally 60 °C for 12 h. Afterwards, test tubes were allowed to cool and newly formed rod like hydrogels were removed with the help of a spatula from the test tubes. The developed hydrogels were sliced into small discs of almost 9 mm length by using a sharp-edged blade. Hydrogel discs were dipped in equimolar contents of water and alcohol (50:50) in order to remove non-reactive agents. After thorough washing, drying of discs was performed in a dry heat oven at 40 °C. After complete drying, dried hydrogel discs were stored in an airtight polyethylene plastic bag for further use [34]. The proposed schematic presentation of HPβCD-grafted-poly (MAA) hydrogels is shown in Figure 14.

### 4.3. Fourier Transform Infrared Spectroscopy (FTIR)

FTIR scans of pure cytarabine, HPβCD, cytarabine-loaded and unloaded hydrogels were recorded to confirm structural integrity and to check for any new complex formation using an FTIR (Perkin Elmer Instrument) spectrophotometer. Scanning limit was 700–4000 cm^−1^.

### 4.4. Thermo-Analytical Investigation

In order to evaluate thermal stability and to determine phase transition temperatures, thermal studies (DSC and TGA) of polymer, pure cytarabine and cytarabine-loaded hydrogels were carried out using a thermo gravimetric analyzer (West Sussex, UK of Q5000 series) under nitrogen stream. A weighed sample loaded in a platinum pan was heated over a temperature range of 0–600 °C [35].

### 4.5. X-ray Diffraction Studies 

PXRD studies were done in order to verify the nature of ingredients and cytarabine within the developed hydrogels. PXRD diffractograms of pure cytarabine, HPβCD with blank and cytarabine-loaded hydrogel were recorded using an X-ray analytical Xpert powder diffractometer in the scanning range of 2*θ* = 10–100° using 3°/min scanning speed [36].

### 4.6. EDX Studies

EDX study was carried out to further confirm loading of cytarabine into the developed network by recording and comparing EDX scans of pure cytarabine, blank hydrogels and cytarabine containing hydrogels through scanning electron microscopy equipped with EDX—an Oxford instrument (UK). 

### 4.7. Scanning Electron Microscopy (SEM)

The surface morphology of prepared hydrogel was studied by SEM using an optical microscope (Vega 3, Tuscan). An optimum sized sample was placed on an aluminum stump coated with a thin layer of gold (~3000 Å) and photomicrographs were collected [37].

### 4.8. Sol-Gel Fraction

Dried hydrogels (1.5 g) were broken down into smaller particles and extraction of water-soluble constituents was performed in boiling water using a Soxhlet apparatus. After a period of 4 h, macro-sized particles were collected on a screen and subjected to drying in a hot air oven.

Gel fraction was measured using the following expression:

$$\mathrm{Gel}\;\mathrm{fraction}\;\%=Wo-\frac{Wi}{Wo}x\;100$$

where, *Wo* = weight before extraction and *Wi* = weight after extraction process [38,39]. Measurements were conducted in triplicate.

### 4.9. Swelling of Hydrogels

The dried hydrogel disc (1 g) was placed in a specific volume of phosphate buffer at room temperature and taken out at predetermined time intervals of 1, 2, 3, 6, 8, 10, 12, 14, 16, 20 and 24 h in order to calculate the equilibrium swelling ratio. Excess surface water was removed using filter paper and hydrogel was weighed again. Measurements were conducted in triplicate.

Equilibrium swelling ratio was determined through using the following expression:

$$Equilibrium\;Swelling\;ratio=W_{f}/W_{i}$$

where, *W_f_* = weight of swollen hydrogel at specific time points and *W_i_* is the weight at dried state [40].

### 4.10. Cytarabine Loading (%)

An accurately weighed amount (1.67 g) of dried hydrogel discs was soaked individually in a 1% (*w/v*) phosphate buffer (pH 7.4) solution of cytarabine until there was no weight change. Swollen hydrogel discs were removed from the solution and rinsed with distilled water to get rid of surface adhered drug. Discs were placed in dry heat oven (Memmert, Schwabach, Germany) at 35 °C for complete drying [41].

### 4.11. Mechanical Strength

A smooth freshly prepared hydrogel was cut and fixed in between mobile and static jaws of the tensile tester equipped with 10 kN load and TIRA software at a speed of 50 mm/min in a controlled environment. Young’s modulus was calculated from the slope of a curve drawn between tensile stress–strain curves. Similarly, stress (*σ*), i.e., force per unit area and strain (*ε*), i.e., changes in length were measured using the following expressions:

$$\sigma =\frac{F}{\pi r^{2}}$$

where, *F* = actual load applied on the hydrogel sample and *r* = radius of the hydrogel and

$$\varepsilon =\frac{lf-li}{li}$$

where, *lf* = final length and *li* = initial length [42].

### 4.12. In Vitro Drug Release Studies and Kinetic Modeling

The pH-dependent release of cytarabine from HPβCD-co-poly (methacrylic acid) hydrogels (1.75 g, equivalent to 500 mg of pure cytarabine) was determined using USP Type II dissolution apparatus. Dissolution media used were 0.1 N HCl (pH 1.2) and phosphate buffer pH 7.4. Each basket was filled with 900 mL of buffer solution. The paddle speed was 50 rpm at temperature of 37 ± 0.5 °C and rotated at 50 rpm. Liquid aliquots (5 mL) were withdrawn at predetermined time intervals up to 2 h and 24 h from the acidic buffer and basic phosphate buffer pH 7.4, respectively. Fresh volume of media was replaced after each withdrawal to maintain sink conditions. Cytarabine content from the developed hydrogel was determined at 281 nm by UV spectrophotometer (UV–1600, Shimadzu, Germany) after filtering through filter paper (0.45 mm, Sartorius) and subsequent filtration. Measurements were conducted in triplicate. Drug release data was processed through zero, first order, Korsemeyer–Peppas, Higuchi and Hixson–Crowell models to explore a suitable kinetic model and to confirm the pattern of cytarabine from the developed network [43,44,45]. 

### 4.13. Acute Oral Toxicity Studies

Healthy male albino rabbits (*n* = 18) having weight ranging from 2.3 to 2.5 kg were used in toxicity experiments. All the procedures adapted for the current study were dually evaluated and approved by the ethics committee of the faculty under its notification No. IREC2020-29. 

Rabbits were kept in bird cages for 7 days with day and night 12 h cycle to acclimatize them with conditions. Rabbits were divided into three groups (*n* = 6), i.e., control (group A), treated (group B) and treated (group C). Rabbits in group B and group C were administered with the developed hydrogel (HPM-3), i.e., 2 and 4 g/kg, respectively, and were observed for two weeks for topical disorders, variation in body mass, consumption of water and food, physical changes, blood chemistry, RFTs, LFTs, AST and ALT levels. Rabbits were restricted from food for up to 10 h before the start of the experiment with free access to water.

At the end of the 14th day, test animals were reweighed and administered anesthesia (1 mL/kg) containing a mixture of ketamine and xylazine (70:30). Hematological samples were withdrawn from the jugular vein and transferred immediately into EDTA tubes. Rabbits were sacrificed and vital organs were removed for histopathological examination, properly washed and transferred to a container having 10% formalin as a preservative solution [36].

### 4.14. In Vivo Pharmacokinetic Evaluation

An in vivo study was performed to compare the pharmacokinetic profile of the prepared cytarabine-loaded hydrogel to that of pure cytarabine using rabbits. For preclinical assessment, the rabbit model was chosen because of its easy handling, more clinically responsive behavior and ease of dose administration. Moreover, multiple samples per rabbit can be withdrawn without any harm to the animal. Healthy male albino rabbits *(n* = 18) having weights ranging from 2 to 2.5 kg were procured from the animal house of the Faculty of Pharmacy and utilized in pharmacokinetic experiments. Eighteen healthy albino male rabbits (2–2.5 kg), divided into three groups (*n* = 6) were used for pharmacokinetic evaluation. A cross-over study design was selected. All the rabbits were deprived of food for at least 12 h prior to dosing while giving free access to water [17]. Pure cytarabine and cytarabine-containing hydrogels (10 mg/kg) were administered orally enclosed in hard gelatin capsules to group A and B rabbits, respectively. Nothing was administered to group C. To quantify cytarabine within plasma, a 3 mL blood sample was collected from the ear vein of rabbits at predefined time points (0, 0.5, 1, 1.5, 2.0, 2.5, 3.0, 4.0, 5.0, 6.0, 9.0, 12.0, 15.0, 18.0, 21.0, 24.0 h), transferred to EDTA tubes and centrifuged at 5000 rpm. Extracted plasma was transferred to Eppendorf tubes and stored at −20 °C in an ultra-low freezer (Sanyo-Tokyo-Japan, −80 °C).

### 4.15. HPLC Estimation of Cytarabine in Plasma

Frozen plasma samples were kept at normal room conditions. To 1 mL of plasma sample, 1 mL of acetonitrile was added to precipitate plasma proteins. The resultant solution was vortexed for 3–5 min and centrifuged at 5000 rpm for 10 min. Supernatant was carefully separated, filtered and diluted with mobile phase. Filtrate (approx. 20 μL) was inserted into injection port of HPLC. Mobile phase, water and acetonitrile (50:50) was operated at a flow speed of 1.2 mL/min. The total run time was 10 min and chromatograms were recorded at 272 nm [46].

### 4.16. In Vitro Degradation Studies

In vitro degradation studies were conducted by the same method used by Gao et al. (2013) with slight modifications. Three liters of HCl buffer (pH 1.2) and phosphate buffer (pH 7.4) were separately prepared as per USP monograph. A measured quantity of pepsin (2.88 g/900 mL) and pancreatin (9 g/900 mL) was incorporated into the HCL buffer and phosphate buffer, respectively, to make artificial gastric fluid (AGF) and artificial intestinal fluid (AIF). For each formulation, 900 mL media was poured into a vessel of dissolution apparatus (USP Type II). Hydrogel discs of known weight were poured into these media separately. Temperature was kept at 37 °C and paddle speed was kept at 50 rpm. Hydrogel discs were removed at predetermined time intervals, i.e., 0, 0.5, 1, 1.5, 2, 3, 4, 6 and 8 h, blotted with absorbant tissue and weight measurements were conducted in order to detect weight lost at each time point [47].

## Figures and Tables

**Table 1:** Cytarabine release kinetic interpretations.

Formulations	Zero Order	1st Order	Higuchi	Korsemeyer–Peppas	
	R^2^	R^2^	R^2^	R^2^	*n*
HPM-1	0.9971	0.9489	0.8798	0.985	0.914
HPM-2	0.9974	0.9443	0.8836	0.985	0.901
HPM-3	0.9979	0.941	0.8824	0.988	0.91
HPM-4	0.9984	0.9805	0.8506	0.9989	1.037
HPM-5	0.9962	0.9808	0.8577	0.9962	1.001
HPM-6	0.9905	0.9665	0.8213	0.9944	1.137
HPM-7	0.9991	0.9615	0.8742	0.9963	0.955
HPM-8	0.9991	0.9545	0.8873	0.9928	0.903
HPM-9	0.9985	0.9513	0.887	0.9939	0.901

**Table 2:** Medical interpretation in acute oral toxicity.

Interpretations	Group A (Control)	Group B (Treated with Developed Hydrogel (2 g/kg)
Signs of illness	Nil	Nil
Weight (kg)		
Pretreatment	2.5 ± 1.1	2.6 ± 1.5
First day	2.11 ± 1.6	2.12 ± 1.3
After 7 days	2.10 ± 2.0	2.14 ± 2.4
After 14 days	2.13 ± 2.2	2.16 ± 2.7
Fluid intake (mL)		
Pretreatment	182.51 ± 2.13	192.10 ± 1.4
First day	194.21 ± 1.5	198.11 ± 1.18
After 7 days	222.13 ± 2.12	206.14 ± 1.31
After 14 days	219.59 ± 1.19	223.21 ± 2.14
Diet intake (g)		
Pretreatment	80.11 ± 1.11	77.61 ± 1.12
First day	87.21 ± 0.07	79.15 ± 1.15
After 7 days	89.51 ± 1.12	83.41 ± 1.21
After 14 days	84.18 ± 1.17	81.33 ± 1.14
Others		
Skin sensitivity	Nil	Nil
Ocular toxicity	Nil	Nil
Death	Nil	Nil

Note: All values are calculated as mean ± SD (*n* = 3).

**Table 3:** Results of hematological analysis of rabbits’ blood.

Parameters	Group 1 (Control)	Group II (Treated with Developed Hydrogel (2 g/kg)
Hemoglobin (g/dL)	12.68 ± 0.23	12.84 ± 0.22
pH	7.29 ± 0.17	7.56 ± 0.15
White blood cells (×10^3^ uL^−1^)	12.16 ± 0.19	12.66 ± 0.27
Red blood cells (×10^6^ uL^−1^)	6.34 ± 0.14	6.42 ± 0.31
Platelets (×103 μ L^−1^)	272 ± 2.5	274 ± 2.1
Monocytes (%)	4.33 ± 0.03	4.41 ± 0.04
Neutrophils (%)	29.26 ± 2.68	27.16 ± 2.11
Lymphocytes (%)	59.29 ± 1.6	57.21 ± 0.41
Mean corpuscular volume (fl)	75.89 ± 2.1	75.62 ± 0.15
Mean corpuscular hemoglobin (pg/cell)	27.17 ± 2.17	26.36 ± 2.7
Mean corpuscular hemoglobin concentration (g/dL)	31.67 ± 1.24	33.12 ± 1.17

Note: All values are given as mean ± SD (*n* = 3).

**Table 4:** Biochemical analysis of HPβCD polymeric hydrogel treated rabbits.

Biochemical Analysis	Group 1 (Control)	Group II (Treated with Developed Hydrogel (2 g/kg)
Urea (mmol/L)	64.48 ± 1.6	64.56 ± 2.2
Creatinine (mg/dL)	1.4 ± 0.16	1.4 ± 0.32
Bilirubin mg/dL	0.96 ± 0.24	0.96 ± 0.26
ALT (IU/L)	66.36 ± 3.06	67.21 ± 1.21
AST (IU/L)	74.61 ± 1.4	74.67 ± 1.31
ALK Phos (IU/L)	23.37 ± 2.33	23.48 ± 2.36

Note: All values are mentioned as mean ± SD *(n* = 3).

**Table 5:** Pharmacokinetic parameters of pure Cytarabine powder solution and Cytarabine loaded hydrogel after oral administration.

Parameters	Cytarabine Oral Powder	Hydrogel Formulation
C_max_ (μg/mL)	4.438	3.85
t_max_ (Hrs)	2	4
AUC_0-24_(μg/mL·h)	15.879	45.359
AUC_0-inf_ (μg/mL·h)	20.093	56.02
AUMC_0-inf_ (μg/mL·h^2^)	129.327	836.86
t_last_ (Hrs)	24	24
t_1/2_ (Hrs)	4.13	8.75
MRT (Hrs)	6.43	14.93
C_last_ (μg/mL)	0.159	0.21
Vz (mg)/(μg/mL)	2.97	2.254
Cl(mg)/(μg/mL) /h	0.497	0.178

Note: All values are given as mean ± SD (*n*=3).

**Table 6:** Composition of HPβCD-grafted-poly (MAA) hydrogels (HPM-1 to HPM-9).

Formulations	HPβCD (g)	Methylene Bisacrylamide (g)	Methacrylic Acid (g)	Ammonium Persulphate (g)
HPM-1	0.60	0.15	15	0.15
HPM-2	0.95	0.15	15	0.15
HPM-3	1.25	0.15	15	0.15
HPM-4	0.65	0.15	15	0.15
HPM-5	0.65	0.17	15	0.15
HPM-6	0.65	0.19	15	0.15
HPM-7	0.65	0.15	17	0.15
HPM-8	0.65	0.15	19	0.15
HPM-9	0.65	0.15	21	0.15
